# Medical Students’ Attitudes towards Overweight and Obesity

**DOI:** 10.1371/journal.pone.0048113

**Published:** 2012-11-05

**Authors:** Birte Pantenburg, Claudia Sikorski, Melanie Luppa, Georg Schomerus, Hans-Helmut König, Perla Werner, Steffi G. Riedel-Heller

**Affiliations:** 1 Integrated Research and Treatment Center (IFB) AdiposityDiseases, University of Leipzig, Leipzig, Germany; 2 Institute of Social Medicine, Occupational Health and Public Health, University of Leipzig, Leipzig, Germany; 3 Department of Psychiatry and Psychotherapy, University of Greifswald, Stralsund, Germany; 4 Department of Medical Sociology and Health Economics, University Medical Center Hamburg-Eppendorf, Hamburg, Germany; 5 Faculty of Social Welfare and Health Sciences, University of Haifa, Haifa, Israel; University of Colorado Denver, United States of America

## Abstract

**Objective:**

Studies from the USA have identified medical students as a major source of stigmatizing attitudes towards overweight and obese individuals. As data from Europe is scarce, medical students’ attitudes were investigated at the University of Leipzig in Leipzig, Germany.

**Design:**

Cross-sectional survey containing an experimental manipulation consisting of a pair of vignettes depicting an obese and a normal weight 42-year-old woman, respectively. Vignettes were followed by the Fat Phobia Scale (FPS), a semantic differential assessing weight related attitudes. In case of the overweight vignette a panel of questions on causal attribution for the overweight preceded administration of the FPS.

**Subjects:**

671 medical students were enrolled at the University of Leipzig from May to June 2011.

**Results:**

The overweight vignette was rated significantly more negative than the normal weight vignette (mean FPS score 3.65±0.45 versus 2.54±0.38, p<0.001). A higher proportion of students had negative attitudes towards the overweight as compared to the normal weight individual (98.9% versus 53.7%, p<0.001). A “positive energy balance” was perceived as the most relevant cause for the overweight, followed by “negligent personality trait”, “societal and social environment” and “biomedical causes”. Attributing a “positive energy balance” or “negligent personality trait” as relevant cause for the overweight was positively associated with negative attitudes.

**Conclusion:**

The results of this study confirm and complement findings from other countries, mainly the USA, and indicate that weight bias in the health care setting may be a global issue. Stigmatizing attitudes towards overweight and obesity are prevalent among a sample of medical students at the University of Leipzig. Negative attitudes arise on the basis of holding the individual accountable for the excess weight. They call for bringing the topic of overweight and obesity more into the focus of the medical curriculum and for enhancing medical students’ awareness of the complex aetiology of this health condition.

## Introduction

The World Health Organisation estimates that in 2008 more than 10% of the world’s adult population were obese [1]. In Germany, approximately 66% of men and 50.6% of women, and 22.6% of boys and 17.7% of girls are overweight or obese according to the International Association for the Study of Obesity [2]. The WHO defines normal weight as a body mass index (BMI, kg/m^2^) of ≥18.5 to <25, overweight as a BMI of ≥25 to <30, and obesity as a BMI of ≥30 [1]. Today, overweight and obesity are the fifth leading risk for global deaths [1]. While overweight and obesity were once considered problems of high-income countries, rapid increases in obesity rates have also been documented in the developing world [3].

Overweight individuals are frequently confronted with weight bias and discrimination [4]. In the USA, perceived stigmatization of overweight individuals has increased by 66% since 1995 and is now comparable to rates of perceived racial discrimination [5]. The media are an important source of promoting, and maintaining as socially acceptable, the stereotypical image of the overweight individual as ugly, stupid and negligent and depict weight as being under one’s own control [6], [7]. Weight bias is present in basically all domains of life: from employment [8], [9] and educational settings [10]–[12] to private life where family and friends are a common source of stigma [13] and overweight may interfere with romantic relationships [14]. Importantly - and worryingly - stigma towards overweight and obese individuals is also common in the health care setting. Medical students and other health care professionals have been identified as a major source of stigmatisation and weight bias: medical students perceived obese patients as less likely to making lifestyle changes, following recommended dietary regimen, responding to counselling and being compliant [15], [16]. Students rated heavier patients as ugly, lazy, sloppy and more depressed [16], [17]. In addition, medical students mentioned morbidly obese patients as a main target of derogatory humour in the hospital setting as patients were perceived to be responsible for their excess weight and caused additional work for the hospital staff [18]. Similarly, physicians would spend less time with heavier patients, view them more negatively, have less respect and were reluctant to perform pelvic examinations among obese women [19]–[21]. Physicians associated obese patients with poor hygiene, non-compliance, hostility and dishonesty and perceived patients as less likely to adhere to medications [22], [23].

In turn, obese patients may delay seeking health care [24] and are less likely to get preventive services, such as cancer screenings [25]–[27]. Obese women may delay preventive care out of fear of disrespectful treatment by health care personnel, negative attitudes of health care providers, embarrassment at being weighed, and medical equipment being too small to be functional [28]. Thus, weight bias in the medical setting and among health care professionals is a major concern and may contribute to sub-optimal health care for overweight and obese patients. As data on medical students’ attitudes towards overweight and obesity stem almost exclusively from the USA and, to our knowledge, work from Europe is lacking attitudes of medical students at the University of Leipzig in Leipzig, Germany were investigated.

## Materials and Methods

### Ethics Statement

The study was approved of by the Ethics committee of the University of Leipzig (Ethik-Kommission an der Medizinischen Fakultät der Universität Leipzig). Before distribution of the questionnaires study participants were verbally informed about the purpose of the study and that participation in the study was completely voluntary. As questionnaires were filled out anonymously no consent form was administered with the questionnaires. Return of a filled questionnaire was taken as consent to participate in the study. The ethics committee specifically approved this procedure.

### Study Population and Study Design

A paper-pencil-survey was conducted among medical students at the University of Leipzig in Leipzig, Germany from May to June 2011. Questionnaires were distributed during lectures and seminars of consenting lecturers in study years 1 through 5. Of the 845 distributed questionnaires 715 were returned. Of these, 2 questionnaires were returned empty, in 17 cases the student stated to be a dental student and in 27 cases the student did not state whether s/he was a dental or a medical student. This left us with 671 questionnaires for data analysis. With a response rate of 84.6% we could analyze the data of 40.3% of students enrolled in the first 5 years of the medical curriculum at the University of Leipzig during the summer semester of 2011, corresponding to 28.9% of the total medical student population (first to 6^th^ year).

### Questionnaire and Experimental Manipulation Using Vignettes

The questionnaire addressed socio-demographics, and general knowledge and opinions about overweight and obesity. A key element of the questionnaire was an experimental manipulation consisting of a pair of vignettes. Questionnaires containing an experimental manipulation are a common method to assess weight stigma [29]. The use of vignettes was adopted from the field of mental health research where they are frequently employed to assess stigmatizing attitudes by describing a hypothetical patient [30]–[32]. Based on the literature, discussions with experts in the field and within focus groups a panel of overweight and normal weight vignettes differing in age and sex were previously developed by our study group. As the literature suggests that women may be more prone to weight-related stigma [33] a pair of adult female vignettes was chosen for the present survey. The first vignette depicted an obese 42-year-old woman: “We would now like to briefly introduce to you a person by way of example: Imagine a woman who is 42 years old and working. At a height of 1.68 m she weighs 90 kilograms, and is thus very overweight. Frequently she has problems to find clothes that fit her. Sometimes climbing stairs is difficult for her and she gets out of breath easily.” The other vignette described an age-matched female individual of normal weight: “We would now like to introduce another person to you: Imagine a woman who is 42 years old and working. At a height of 1.68 m she weighs 62 kilograms, and is thus of normal weight. She never has problems to find clothes that fit her. Climbing stairs is easy for her and she is physically enduring.”

Weight and height measurements corresponded to a BMI of 32 kg/m^2^ and 22 kg/m^2^, respectively. The overweight vignette was presented first, followed by a panel of questions on causal attribution for the woman’s excess weight, and then a semantic differential assessing participants’ weight related attitudes. Then, the normal weight vignette was introduced, followed by the semantic differential.

### Causal Attributions

To assess participants’ causal attributions regarding the depicted woman’s overweight 14 potential causes were presented. These items were developed based on previous qualitative focus group research [34]. Participants were asked to rate the relevance of potential causes for the woman’s overweight on a 5-point Likert scale ranging from “1 = not relevant at all” to “5 = extremely relevant”. In order to identify underlying themes factor analysis was performed. As underlying causes were considered to potentially correlate with each other promax rotation was applied. Kaiser Criterion of eigenvalues >1 was used and items with factor loadings ≥0.5 were included in the analysis. A mean relevance score was calculated for each factor, excluding participants with more than 5 missing values.

### Weight-related Attitudes - Semantic Differential

Stigmatizing attitudes were assessed using the short form of the Fat Phobia Scale (FPS) by Bacon et al. [35]. The scale was translated into German, following TRAPD (translation, review, adjudication, pre-testing and documentation) guidelines [36]. Pre-testing was done in qualitative focus groups. The FPS consists of 14 pairs of adjectives on a semantic differential (e.g. industrious - lazy). The participant is asked to select the attribute that in his/her opinion closest describes the individual in the vignette on a scale ranging from 1 to 5, where “1” stands for the first adjective (e.g. industrious) and “5” for its opposite (e.g. lazy). The scale was recoded such that a higher score reflects a more negative view of the vignette. A mean FPS score was calculated excluding participants with more than 5 missing values.

As suggested by Puhl et al. FPS scores were categorized into “more positive or neutral attitudes” (scores ≤2.50) and “more negative attitudes” (scores >2.50) [37]. Mean score and internal consistency of the translated FPS version for the overweight vignette were comparable to those of the original FPS.

### Data Analysis

Comparison of FPS score means was performed using Wilcoxon signed-rank test and comparison of proportions was done by χ^2^ test. In all analyses “no response” codes were treated as missing values. Linear regression was conducted to determine which variables correlated with students’ attitudes towards overweight and obesity. The mean FPS score for the overweight vignette was therefore used as dependent variable in this model. Participants’ BMI (calculated from self-reported weight and height measurements), age, attributed causes for overweight derived from factor analysis, and whether the participant considered the individual or the society responsible for finding a solution to the obesity problem (lower scores indicate higher responsibility of the individual) were introduced as continuous variables. Sex, migrational background (participant not born in Germany or not in possession of a German passport, or at least one of participant’s parents not born in Germany, definition adopted from Federal Statistical Office of Germany [38]), whether the participant had ever experienced or not experienced weight bias, and whether the participant had ever had contact or never had contact with overweight or obese patients were dummy coded before introducing them into the regression model. To assess systematic rating tendencies the mean FPS score for the normal weight vignette was also included as continuous independent variable. Participants with missing values on independent variables were excluded and the linear regression was performed with 573 individuals. All data analysis was conducted with Stata 10.0.

## Results

### Characteristics of the Study Population

The study population consisted of 671 medical students from the 1^rst^ to the 5^th^ year. The characteristics of the study population are detailed in Table 1. Percentage of women did not differ between our sample and the total medical student population at that time (67.3% versus 64.2%, p = 0.177). However, the mean age of the sample was significantly younger than that of the total student population (23.1 versus 26 years, p<0.001). Of the study participants 84% were of normal weight and approximately 10% were either overweight or obese. The majority (61.9%) of the students were in the clinical part of their studies. The percentage of women who stated that they had previously experienced some sort of weight bias was more than twice as high as that of men (24.0% versus 11.6%, respectively).

**Table 1:** Socio-demographics of the study population (n = 671).

	N (%) or Mean (± SD, range)	Women	Men
Female	450 (67.3)		
Mean age (years)	23.1 (±2.88, 18–45)		
Migrational background*	70 (10.5)		
Clinical part	415 (61.9)		
Mean BMI	22.00 (±2.64, 17.36–34.95)	21.49 (±2.61, 17.36–34.95)	23.02 (±2.42, 17.92–33.95)
Underweight	35 (5.5)	32 (7.6)	3 (1.4)
Normal weight	534 (84)	359 (85.1)	174 (81.7)
Overweight	60 (9.4)	26 (6.2)	34 (16)
Obese	7 (1.1)	5 (1.2)	2 (0.9)
Experienced weight bias	130 (19.9)	105 (24)	25 (11.6)
Contact with ow patients	573 (86.3)		

SD = standard deviation; ow = overweight.

* definition of migrational background adopted from Federal Statistical Office of Germany: Participant not born in Germany or not in possession of German passport, or at least one of participant’s parents not born in Germany [38].

### General Knowledge on Overweight and Obesity

Of all participants 49.8% agreed “strongly” and 38.7% “somewhat” to the statement that “overweight is one of the most important health problems in Germany today”. Similarly, 67.4% agreed “strongly” and 26.3% “somewhat” that “overweight increases the risk for other diseases such as diabetes and cancer”. Students estimated that participants of a weight-loss programme would lose an average of 12.1% (±9.7, range 0–85%) of their body weight within 6 months. Students believed that 18.8% (±15.5, range 0–95%) of participants could keep their reduced weight over a long-term period of time.

When asked about their opinion on whether finding a solution for the overweight epidemic was the responsibility of the society or the individual, 43.5% of participants stated that it was “rather” the individual’s responsibility while 44.1% of participants stated that it was equally the individual’s and the society’s responsibility.

### Causal Attributions

Following the introduction of the overweight vignette students’ opinions regarding the relevance of a panel of potential causes for the woman’s overweight were assessed. Factor analysis identified four underlying themes (Table 2): Factor 1 was labelled “negative influence of societal and social environment” (misleading advertisement and product labelling, cultural influences, social environment, affluence of food in our society). Factor 2 was summed up as “positive energy balance” (lack of physical activity, quality of food, too much food) while factor 3 was labelled “negligent personality trait” (boredom, lack of willpower). Finally, factor 4 was named “biomedical causes” (endocrine and metabolic disorders, genetic factors).

**Table 2:** Causal attributions – factor analysis.

	Factor loading*			
Potential Cause of Overweight and Obesity	Factor 1	Factor 2	Factor 3	Factor 4
Societal and Social Environment				
Misleading advertisement/product labelling	0.79			
Cultural influences	0.68			
Social environment	0.63			
Affluence of food	0.59			
Lack of knowledge about nutrition	0.44	0.38		
Child-rearing errors	0.40		0.4983	
Energy Balance				
Lack of physical activity		0.82		
Quality of food		0.76		
Too much food		0.65	0.30	
Personality Trait				
Boredom			0.75	
Lack of willpower			0.73	
Psychological problems			0.47	0.41
Biomedical causes				
Endocrine and metabolic disorders				0.84
Genetic Factors				0.82

* Items with factor loadings ≥0.5 were included, however factor loadings ≥0.3 are shown as well.

A mean relevance score was calculated for each factor (Table 3). A “positive energy balance”, including the aspects “lack of physical activity” and “too much food”, was rated as the most relevant cause for the woman’s overweight, followed by “negligent personality trait” and “negative influence of the societal and social environment”. “Biomedical causes” received the lowest relevance score. When comparing the perceived causes amongst each other, “positive energy balance” was rated as significantly more relevant than all other causes (“positive energy balance” versus “negligent personality trait”, z = 18.890; “positive energy balance” versus “negative influence of the societal and social environment”, z = 19.360; and “positive energy balance” versus “biomedical causes”, z = 18.351, in all cases p<0.001). “Negligent personality trait” was rated as significantly more relevant than “biomedical causes” (z = 2.290, p = 0.022) for the development of overweight.

**Table 3:** Causal attributions – perceived relevance of factor as cause of obesity.

	Mean relevance score (± SD)
**Energy Balance**	
Lack of physical activity	4.42±0.84
Too much food	4.18±0.90
Quality of food	4.07±1.00
Total score sub-scale “energy balance”	4.23±0.72
**Personality Trait**	
Lack of willpower	3.51±1.01
Boredom	3.01±1.10
Total score sub-scale “personality trait”	3.26±0.87
**Societal and Social Environment**	
Affluence of food	3.60±1.12
Social environment	3.48±1.01
Misleading advertisement and product labelling	3.10±1.16
Cultural influences	2.70±1.08
Total score sub-scale « environment »	3.22±0.79
**Biomedical causes**	
Genetic Factors	3.19±0.97
Endocrine and metabolic disorders	3.12±1.09
Total score sub-scale “biomedical”	3.15±0.89
**Total score**	3.48±0.52

Participants were to chose from a 5-point Likert scale ranging from 1 = ”not relevant at all” to 5 = ”extremely relevant”; SD = standard deviation.

### Weight-related Attitudes

Students’ weight-related attitudes were then assessed using the FPS. The mean FPS score for the overweight vignette was significantly higher than that for the normal weight vignette (3.65±0.45 versus 2.54±0.38, z = 21.438, p<0.001). Thus, the overweight vignette was rated significantly more negative than the normal weight vignette.

When FPS scores were split into the categories “more positive or neutral attitudes” (scores ≤2.50) and “more negative attitudes” (scores >2.50), a significantly higher percentage of students had negative attitudes towards the overweight individual as compared to the normal weight individual (98.9% versus 53.7%). Consistently, a significantly lower percentage of students had positive attitudes towards the overweight individual as compared to the normal weight individual (1.1% versus 46.3%, χ^2^ test = 369.7882, p<0.001). 4.13% of the participants exhibited a high level of fat phobia (FPS score ≥4.4) towards the overweight vignette.

Linear regression was then performed using the mean FPS score for the overweight vignette as dependent variable (Table 4). Attributing a “positive energy balance” or “negligent personality trait” as relevant causes for overweight was positively associated with negative attitudes towards the overweight vignette. Also, the more a participant considered the individual responsible for finding a solution to the obesity problem, the more negative his/her attitudes were. In contrast, being of female sex was associated with less negative attitudes. Finally, a more negative rating of the normal weight vignette was associated with a more positive rating of the overweight vignette. However, whether participants attributed the cause of overweight to the “negative influence of societal and social environment” or “biomedical causes” was not associated with attitudes towards the overweight vignette. Also, participants’ BMI, age, migrational background, own experience of weight bias or previous contact with overweight patients were not associated with weight related attitudes. Correlations among variables were weak (correlation coefficients <0.3, data not shown), with the exception of moderate correlations between “mean FPS score for normal weight vignette” and “mean FPS score for overweight vignette” (correlation coefficient: −0.47, p<0.001); “negligent personality trait” and “positive energy balance” (correlation coefficient: 0.33, p<0.001); “negative influence of societal and social environment” and “negligent personality trait” (correlation coefficient: 0.32, p<0.001). This model explained 27% of the total variance.

**Table 4:** Variables associated with stigmatizing attitudes of students towards overweight.

Variable	Coefficient (SE)	95% Confidence interval	p
Positive energy balance	0.0932 (0.0241)	0.0458–0.1406	<0.001
FPS score for normal weight	−0.5121 (0.0446)	−0.5997– −0.4246	<0.001
Female sex	−0.0968 (0.0373)	−0.1701– −0.0235	0.01
Finding solution is responsibility of individual or society^a^	−0.0530 (0.0235)	−0.0991– −0.0069	0.024
Personality trait	0.0447 (0.0209)	0.0036–0.0859	0.033
Biomedical causes	−0.0339 (0.0187)	−0.0706–0.0028	0.07
Age	−0.0094 (0.0061)	−0.0213–0.0026	0.124
Previous contact with overweight patients	−0.0604 (0.0503)	−0.1592–0.0384	0.230
Environmental influence	0.0178 (0.0229)	−0.0272–0.0628	0.438
Experience of weight-bias	0.0276 (0.0435)	−0.0579–0.1131	0.527
BMI	0.0015 (0.0066)	−0.0115–0.0144	0.823
Migrational background	0.0021 (0.0560)	−0.1079–0.1121	0.971

SE = standard error.

a Participants were asked on a 5-point Likert scale whether they perceived the individual ( = 1) or the society ( = 5) as responsible for finding a solution for the obesity epidemic (lower scores indicate higher perceived responsibility of the individual).

## Discussion

Studies mainly conducted in the USA have identified medical students as an important source of weight bias and weight related stigma [15]–[18]. To our knowledge, data from Europe however is lacking. Crandall et al. compared weight related attitudes among various different nations, including the USA [39], [40]. Anti-fat attitudes differed between different countries and findings from the USA could not be generalized to other countries. The aim of the present study was therefore to start filling the gap in knowledge and to investigate medical students’ attitudes towards overweight and obesity at the University of Leipzig in Leipzig, Germany.

Nearly 90% of the enrolled medical students agreed that overweight is one of Germany’s pressing health problems today and that it increases the risk for other diseases. Therefore, apparently, medical students are aware of the urgency of this health condition. At the same time, however, stigmatizing attitudes towards obese individuals were prevalent among the study population.

Medical students’ negative attitudes towards overweight and obesity were comparable to those reported after employing the original short form of the FPS developed by Bacon et al. (3.65 versus 3.7 in a sample from 1984–1991 and 3.6 in a sample from 1999) [35]. Interestingly, we found a similar FPS score in a recent telephone survey among the German general population (3.62±0.51, Sikorski et al. submitted). Also, the percentage of medical students exhibiting negative attitudes towards obese individuals (98.9%) was very similar to that found among the German general population (98.8%) (Sikorski et al. submitted). Bacon (2001) interprets a score of 3.6 as an indication of an average amount of fat phobia, while a score of 4.4 or above indicates a high level of fat phobia [35]. Thus, our study population expresses an average amount of stigmatizing attitudes towards overweight and obesity. While it is somewhat reassuring that most medical students do not express a high level of stigmatizing attitudes it is still surprising that medical students express a level of stigma similar to that of the general population. One should assume that individuals who receive comprehensive medical training and whose goal it is to work with a variety of patients would express a lower level of stigmatizing attitudes. Indeed, one study measuring implicit weight bias suggests that physicians may have less stigmatizing attitudes than the general population [41]. However, the findings of that study have to be interpreted with caution due to differences among the two samples in that study. Worryingly, our findings support previous data showing that weight bias is pervasive in the health care setting, even among health professionals whose scientific or clinical work focuses on obesity [41], [42].

Stigma and social distance arise when a health condition is perceived as being caused by the afflicted individual’s own behaviour and the person therefore as responsible for his/her unfavourable condition [43]. Body weight is commonly perceived as under the control of the individual [39], [40], [44] and subsequently overweight persons are frequently blamed for their excess weight [4], [45]. Consistently, of the potential causes for overweight specified in the present study only “positive energy balance” and “negligent personality trait” were positively associated with negative attitudes. Both can be perceived as internal factors and, at least to some extent, as under the control of the individual. In line with that, participants who tended to be of the opinion that finding a solution for the obesity epidemic was rather the responsibility of the individual as opposed to the society had more negative attitudes towards excess weight.

A “positive energy balance”, consisting of the components “too much food, lack of physical activity, quality of food” was rated as the most relevant cause for the development of overweight, followed by “negligent personality trait”, “societal and social environment” and lastly “biomedical causes”. While overweight and obesity are the result of a “positive energy balance” the development of this positive energy balance is multifactorial. Sharma and Padwal stress the importance of the question of *why* energy intake exceeds energy consumption [46]. They propose to regard obesity as a disease with a complex aetiology and a wide variety of causes that need to be addressed specifically and individually. One important component in the development of overweight and obesity is the individual’s genetic predisposition [47]. Albeit rated as least important, study participants considered biomedical factors, consisting of genetical factors and endocrine diseases, as one relevant cause for excess weight. In fact, genetic predisposition plays a powerful role in the regulation of appetite, hunger and satiey, and ultimately food intake [48].

That “negligent personality trait” was perceived as the second most important cause for the development of overweight together with the fact that it was associated with more negative attitudes is consistent with the common stereotype that overweight individuals lack self-discipline and are lazy [45]. This image is strongly supported by the media where overweight characters are often ridiculed and the notion reinforced that weight-loss is easily achievable if one were only motivated enough [7], [49].

Interestingly, in the factor analysis we conducted, the item “psychological problems” as a potential cause for overweight loaded both on “negligent personality trait” and “biomedical factors”. However, loading was low and did not meet our cut-off. Biomedical conditions, such as depression, may contribute to the development of overweight [50]. The fact that “psychological problems” also loaded on “negligent personality trait” may indicate students’ indecision about which role psychological problems play in the development of overweight. It may also be an indication for potential stigma among medical students towards psychological illness.

Female participants had less negative attitudes towards the obese individual. This is consistent with findings from several previous studies [44], [51]. Latner et al. speculate whether women may be generally more accepting of obesity than men [51]. However, in contrast, a study by Schwartz et al. revealed that women had higher levels of implicit anti-fat bias [42]. Further research is needed to determine the role of gender in holding explicit and implicit weight related attitudes.

To account for potential systematic rating tendencies among study participants the FPS score for the normal weight vignette was included in the regression analysis. The observation that a more negative rating of the normal weight vignette was significantly associated with a more positive rating of the overweight vignette can be interpreted as evidence for error of central tendency among participants, namely to rate the two vignettes similarly (i.e. towards the “3” on the FPS scale, ranging from “1” ”(i.e. positive) to “5” (i.e. negative).

Interestingly, own BMI and previous contact with overweight patients were not associated with participants’ attitudes. One explanation for the lack of an association may have been insufficient sample size as over 80% of participants had a normal BMI or reported previous contact with overweight patients. However, the current literature is inconclusive about whether own BMI is associated with attitudes towards overweight [40]–[42], [51]. In contrast, research in the field of mental health revealed that contact with mentally ill persons may reduce social distance and stigma [52], [53]. Also, contact with an afflicted person, an “expert by experience”, is considered the key ingredient of successful anti-stigma interventions [54]. However, participants in the present study most likely came into contact with overweight patients in the hospital or a similar setting. There, patients are usually not perceived as “experts” and such contact may not be appropriate for reducing stigmatizing attitudes. One might even argue that health care personnel’s negative attitudes may in part be due to challenges associated with caring for obese patients: conducting physical examinations or diagnostic imaging may be more difficult [55], the peri-operative risk increased [56], the extra weight may pose a special physical strain on health care personnel and functional equipment may often be lacking. However, during the medical curriculum in Germany, students are only minimally involved in patient care, with the exception of final year students, and those were not included in the study (see limitations). It is therefore highly questionable whether medical students’ negative attitudes are primarily based on experiences made while directly caring for overweight or obese patients. Instead, the results of this study raise the question of why the medical curriculum apparently fails to improve medical students’ attitudes towards overweight and obesity. They highlight the importance for bringing the topic of overweight and obesity more into the focus of the medical curriculum, for better educating medical students’ about the complex aetiology of this health condition, and for raising medical students’ awareness of the particular needs of this growing patient population.

This study has several limitations: in the setting of this study it was not feasible to present vignettes in a random manner. However, to minimize bias when assessing attitudes towards excess weight the overweight vignette was presented first. While the collected sample was comparable to the total student population regarding the distribution of men and women, the mean age of the sample was significantly younger than that of the total student population. This is probably due to the fact that we did not enroll students in their final year as they work full time in different teaching hospitals in Leipzig, its surroundings, and abroad. We did not employ male vignettes and vignettes of different age groups. Also, due to the study design we have to rely on self-reported data of participants’ explicit attitudes and cannot rule out that answers were biased by social desirability. However, the experimental set-up “vignette followed by semantic differential” was chosen precisely because it is a more indirect way to assess attitudes as compared to, e.g. more direct approaches were study participants are asked to agree or disagree to blunt statements about overweight people in general [57]. Also, anti-fat attitudes seem to be one of the few stigmatizing opinions that people feel they can freely express [44]. Taken together, we believe that we may rather underestimate the real extent of negative attitudes towards overweight individuals among our sample of medical students.

Weight stigma in the health care setting is detrimental: patients may forego important preventive services and necessary treatment out of fear of disrespectful treatment [24], [28]. On top of that, experiencing weight bias and stigma may increase the risk for psychological distress, feelings of loneliness, depression, poor body image, disordered eating, not engaging in physical exercise [58]–[60], and may therefore even contribute to gaining weight. Medical students constitute the next generation of physicians and their attitudes will influence the health care - and health - of patients today and in the future. It would be utterly counterproductive if medical students contributed to patients’ physical and psychological ailments rather than promoting their health.

### Conclusions

The results of this study confirm and complement findings from other countries, mainly the USA, and indicate that weight bias in the health care setting may be a global issue. They demonstrate that stigmatizing attitudes towards overweight and obesity are prevalent among a sample of German medical students. These negative attitudes arise on the basis of holding the individual accountable for the excess weight. A positive energy balance, consisting of the aspects too much food, bad quality of food and too little exercise, was perceived as the most relevant cause for the development of overweight. These data highlight the importance for bringing the topic of overweight and obesity more into the focus of the medical curriculum, for better educating medical students about the complex aetiology of this health condition, and for raising medical students’ awareness of the particular needs of this growing patient population. Further research to elucidate medical students’ attitudes towards overweight and obese patients is urgently needed.
